# The association of vitamin D, semen parameters, and reproductive hormones with male infertility: A cross-sectional study

**DOI:** 10.18502/ijrm.v20i4.10905

**Published:** 2022-05-23

**Authors:** Leila Maghsoumi-Norouzabad, Maryam Labibzadeh, Ahmad Zare Javid, Seyed Ahmad Hosseini, Gholam Abbas Kaydani, Maryam Dastoorpur

**Affiliations:** ^1^Student Research Committee, Ahvaz Jundishapur University of Medical Sciences, Ahvaz, Iran.; ^2^Center of Therapy and Research Infertility Jahad Daneshgahi, Ahvaz, Iran.; ^3^Department of Nutrition, School of Allied Medical Sciences, Ahvaz Jundishapur University of Medical Sciences, Ahvaz, Iran.; ^4^Nutrition and Metabolic Diseases Research Center, Ahvaz Jundishapur University of Medical Sciences, Ahvaz, Iran.; ^5^Health Research Institute, Infectious and Tropical Diseases Research Center, Ahvaz Jundishapur University of Medical Sciences, Ahvaz, Iran.; ^6^Department of Biostatistics and Epidemiology, Air Pollution and Respiratory Diseases Research Center, Ahvaz Jundishapur University of Medical Sciences, Ahvaz, Iran.

**Keywords:** Infertility, Male, Vitamin D, Semen analysis, Gonadal hormones, Pituitary hormones.

## Abstract

**Background:**

The prevalence of vitamin D deficiency and male infertility is high in Iran.

**Objective:**

The present research aimed to examine the association between serum vitamin D [25(OH)D], parameters of semen including semen volume, sperm count, motility and morphology, and reproductive hormones in infertile Iranian men.

**Materials and Methods:**

This was a cross-sectional study on 119 infertile men conducted between September 2018 and May 2019. Subjects were divided into 3 groups based on serum vitamin D levels: deficient ( < 10 ng/mL), insufficient (10 ng/mL ≤ 25(OH)D ≤ 30 ng/mL), and sufficient ( > 30 ng/mL). Body mass index and waist circumference were measured. 25(OH)D, testosterone, sex hormone-binding globulin, luteinizing hormone (LH), follicle-stimulating hormone and estradiol levels, and semen parameters were assessed.

**Results:**

The semen volume, sperm counts, total and progressively motile sperm, normal sperm morphology, testosterone levels, and testosterone/estradiol ratio were substantially higher in the sufficient group compared to the other groups (p < 0.001). Also, in the sufficient group, serum LH (p < 0.001) and estradiol (p < 0.001, p = 0.01) were notably lower and serum sex hormone-binding globulin (p < 0.001) and the testosterone/LH ratio (p < 0.001) were considerably higher compared to the insufficient and deficient groups.

**Conclusion:**

Our study showed a positive relationship between serum vitamin D levels, and seminal parameters and sex hormones in Iranian infertile males.

## 1. Introduction

Vitamin D deficiency is a common serious public health challenge worldwide (1). It has been considered serum 25-hydroxy vitamin D (25[OH]D) < 50 nmol/l (20 ng/ml) as deficient, 25(OH)D between 50-75 nmol/l (20-30 ng/ml) as insufficient, and a serum 25(OH)D > 75 nmol/l (30 ng/ml) as a sufficient level (2). The main source of this vitamin is its synthesis in the skin through sunlight exposure (1). It has been reported that the existence of vitamin D receptors and its metabolizing enzymes in the prostate, testis, ejaculated spermatozoa, and Sertoli cells may play some significant roles in spermatogenesis and maturation of spermatozoa. In some cross-sectional studies, 25(OH)D deficient men were reported to have lower sperm motility, total numbers of motile sperm, sex hormone-binding globulin (SHBG), testosterone/estradiol ratio, and free androgen index (FAI) compared to 25(OH)D sufficient male subjects (3, 4). On the other hand, some studies have had results that contradict these, reporting no significant relationship between 25(OH)D and parameters of semen and sex hormones (1, 5, 6).

Therefore, the association between vitamin D and male reproductive function remains unclear. The link between male infertility and vitamin D should be studied, especially in countries with high prevalence rates of vitamin D deficiency and male infertility like Iran. The infertility rate in Iranian couples has been reported to be 24.9%, which is higher than the global average (7). If the positive relationships between vitamin D deficiency and infertility parameters are confirmed, vitamin D as a simple, non-aggressive and cheap clinical tool could be used in future infertility treatment and assisted reproductive technologies.

One limitation of the studies that have examined vitamin D is the variability in the vitamin D reference ranges and authors' definitions of vitamin D status, which can consequently affect the obtained results. In most of these studies, serum levels of 25(OH)D < 20 ng/mL have been considered as deficient (8). It is possible that vitamin D could be more influential on semen parameters under conditions with severe vitamin D deficiency (25[OH]D < 10 ng/ml) compared to conditions with mild vitamin D deficiency or insufficiency. Accordingly, this may be the reason that previous studies performed on both men and animals have linked impaired male fertility with severe vitamin D deficiency (4). So, in contrast to the methods of most previous studies, in the present study, we considered serum levels of 25(OH)D < 10 ng/ml as deficient.

Hence, in this study, we investigated the relationship between serum 25(OH)D, and semen parameters and sex hormones with 3 different categories of serum vitamin D status, namely deficient ( < 10 ng/mL), insufficient (10-30 ng/mL) and sufficient ( > 30 ng/mL) in infertile Iranian men (2).

## 2. Materials and Methods

### Study population

This cross-sectional study was conducted on 119 infertile men (ranged between 20-50 yr) who were referred to Jahad Daneshgahi Fertility Clinic in Ahvaz, Iran, from September 2018 to May 2019. A demographic questionnaire was completed for the selected participants. The inclusion criteria were as follows: a history of infertility for at least 1 yr despite having unprotected intercourse, idiopathic infertility, accessibility of the Ahvaz Jahad Daneshgahi infertility clinic, and having no infertile female partner. The men were excluded if they had a history of medical therapy in the past 12 or fewer wk, a history of any systematic or chronic disease, drug or alcohol abuse, or occupational or environmental exposure to possible reproductive toxins. A standard stadiometer was used to measure the height while the participants were standing with no shoes. Weight was measured with light clothing. Body mass index (BMI) was determined as weight (kg)/height (m^2^) (9).

### Semen collection and analysis

Semen samples were taken from the included participants after 2-7 days of sexual abstinence, and these were then transferred immediately to the laboratory. These were analyzed in terms of semen volume, sperm count, progressive and total sperm motility, and normal sperm morphology parameters according to the World Health Organization 2010 criteria (10). The examination of the semen samples was done using the methods described in our previous article (9) by trained clinical technicians.

### Blood sampling and analysis methods

A 5 mL blood sample was taken from subjects in the morning and then was sent to the laboratory to assess serum 25(OH)D, testosterone, follicle-stimulating hormone, luteinizing hormone (LH), SHBG and estradiol. Participants were then divided by 25(OH)D levels into sufficient (25[OH]D > 30 ng/ml, n = 26), insufficient (25[OH]D 20-30 ng/ml, n = 65) and deficient (25[OH]D 30 ng/ml, n = 28) (2).

### Ethical considerations

The Ethics Committee of the Ahvaz Jundishapur University of Medical Sciences, Ahvaz, Iran approved this study (Code: IR.AJUMS.REC.1398.313) and it was carried out in accordance with the Declaration of Helsinki. An informed consent form was signed by all participants.

### Statistical analysis

The Statistical Package for the Social Sciences (SPSS) version 22 (SPSS Inc., Chicago, IL, USA) was used to analyze the data. The median (range), mean ± standard deviation, or number (percentage) were used to present the data. The Kolmogorov–Smirnov statistical test was used to test for normality. One-way analysis of variance (ANOVA) and the Kruskal–Wallis test were used to compare the means of groups to determine any significant differences. The Chi-square test was also used for testing relationships between categorical variables. Moreover, post hoc pairwise comparisons (for variables with normal distribution) and the Mann-Whitney test (for variables without normal distribution) were used to determine differences between means. P-values < 0.05 were considered statistically significant.

## 3. Results

### Demographic and clinical characteristics

A total of 119 infertile men were assessed including 26 (21.84%) men with 25(OH)D > 30 ng/ml (sufficient), 65 (54.62%) cases with 25(OH)D 20-30 ng/ml (insufficient) and 28 (23.52%) with 25(OH)D < 20 ng/ml (deficient). There were no significant differences in age, waist circumference, physical activity, duration of abstinence, infertility duration, smoking, and economic situation between the groups. However, the mean BMI and serum levels of 25(OH)D were significantly different between the 3 groups. The pairwise comparison showed that the mean BMI was substantially lower in the sufficient 25(OH)D group in comparison to the insufficient 25(OH)D group (Table I).

### Semen analysis

Sperm volume and count, total and progressively motile sperm and normal sperm morphology were significantly different between groups. The pairwise comparison showed that sperm volume, sperm count, motile sperm and normal sperm morphology were considerably higher in the sufficient 25(OH)D group in comparison to the insufficient and deficient 25(OH)D groups, and in the insufficient 25(OH)D group in comparison to the deficient 25(OH)D group (Table II).

### Hormone analysis

Serum levels of LH, SHBG, testosterone, the testosterone/estradiol ratio, the testosterone/LH ratio, and estradiol were significantly different between the 3 groups. The pairwise comparison showed that serum LH and estradiol were substantially lower and serum SHBG and the testosterone/LH ratio were considerably higher in the sufficient 25(OH)D group compared to the insufficient and deficient 25(OH)D groups. Also, serum levels of testosterone and the testosterone/estradiol ratio were significantly higher in the sufficient 25(OH)D group compared to the insufficient and deficient 25(OH)D groups, and in the insufficient 25(OH)D group compared to the deficient 25(OH)D group (Table III).

**Table 1:** Characteristics of study population according to the different 25(OH)D groups (n = 119)


[2.2in,lr]**Variables** **25(OH)D (ng/ml)**	**Deficient (< 10) (n = 28)**	**Insufficient (10-30) (n = 65)**	**Sufficient (> 30) (n = 26)**	**P-value**
Age (yr)*	34.32 ± 5.27	34.35 ± 8.00	36.03 ± 6.58	0.62 ${}^{a}$
BMI (kg/m^2^) ${}^{*}$	27.23 ± 5.52	29.12 ± 5.05 ${}^{e}$	26.57 ± 2.60 ${}^{f}$	0.01 ${}^{b}$
WC (cm)*	94.85 ± 12.17	100.38 ± 12.76	95.19 ± 7.05	0.09 ${}^{a}$
Smoking**		
** No**	19 (67.9)	42 (64.6)	20 (76.9)	
** Yes**	9 (32.1)	23 (35.4)	6 (23.1)	0.52 ${}^{c}$
Duration of abstinence (day)*	3.00 ± 0.46	3.18 ± 0.53	3.08 ± 0.32		0.48 ${}^{d}$
Infertility duration (yr) ${}^{\wedge }$ *	4.57 ± 3.96	4.33 ± 5.68	5.92 ± 6.29	0.42 ${}^{a}$
25(OH)D3 (ng/ml)*	9.13 ± 0.99 ${}^{g}$	21.21 ± 5.23 ${}^{h}$	41.20 ± 11.45 ${}^{i}$	< 0.001 ${}^{a}$
*Data presented as Mean ± SD. **Data presented as n (%). BMI: Body mass index, WC: Waist circumference, 25(OH)D: 25-hydroxy vitamin D. Differences between the 3 groups were tested using the Kruskal-Wallis test (a), Welch and Brown-Forsythe (b), Chi-Square test (c), One-way analysis of variance (ANOVA) (d). Dunnett T3Post hoc test, e & f (p = 0.01), g & h & i (p < 0.001), h & i (p < 0.001). ${}^{\wedge }$ Median ± interquartile range (IQR) for infertility duration: 4 ± 5, 2.5 ± 3.5, 2.5 ± 8.75 yr respectively. P < 0.05 was considered statistically significant				

**Table 2:** Sperm analysis results for the different 25(OH)D groups (n = 119)


[2.2in,lr]**Variables** **25(OH)D (ng/ml)**	**Deficient (< 10) (n = 28)**	**Insufficient (10-30) (n = 65)**	**Sufficient (> 30) (n = 26)**	**P-value**	**Pairwise comparison (Mann-Whitney test)**
** Volume (ml)**	1.47 ± 0.77 ${}^{b}$	3.39 ± 0.90 ${}^{c}$	5.46 ± 0.88 ${}^{d}$	< 0.001 ${}^{a}$	b & c (p < 0.001) d (p < 0.001) c & d (p < 0.001)
** Sperm concentration (M/mL)**	7.92 ± 3.82 ${}^{b}$	19.16 ± 3.62 ${}^{c}$	39.03 ± 9.30 ${}^{d}$	< 0.001 ${}^{a}$	b & c (p < 0.001) d (p < 0.001) c & d (p < 0.001)
** Motile sperm (%)**	29.67 ± 3.52 ${}^{b}$	38.50 ± 4.02 ${}^{c}$	62.73 ± 8.4 ${}^{d}$	< 0.001 ${}^{a}$	b & c (p < 0.001) d (p < 0.001) c & d (p < 0.001)
** Progressive sperm (%)**	19.00 ± 5.91 ${}^{b}$	28.60 ± 3.84 ${}^{c}$	59.76 ± 5.56 ${}^{d}$	< 0.001 ${}^{a}$	b & c & d (p < 0.001) c & d (p < 0.001)
** Normal sperm morphology (%)**	2.57 ± 0.57 ${}^{b}$	3.13 ± 0.99 ${}^{e}$	4.92 ± 0.97 ${}^{d}$	< 0.001 ${}^{a}$	b & e (p < 0.001) & d (p < 0.001) e & d (p < 0.001)
Data presented as Mean ± SD. M/ml: Million per milliliter, 25(OH)D: 25-hydroxy vitamin D, Differences between the 3 groups were tested using Kruskal-Wallis test (a). Pairwise comparison was tested using the Mann-Whitney test. P < 0.05 was considered statistically significant					

**Table 3:** Hormone analysis results for the different 25(OH)D groups (n = 119)


[2.2in,lr]**Variables** **25(OH)D (ng/ml)**	**< 10 (n = 28)**	**10-30 (n = 65)**	**> 30 (n = 26)**	**P-value**	**Post hoc tests**
** LH (ng/ml)**	8.18 ± 3.57 ${}^{i}$	8.67 ± 3.75 ${}^{d}$	5.36 ± 2.62 ${}^{j}$	< 0.001 ${}^{a}$	i & j (p < 0.001) d & j (p < 0.001)
** FSH (ng/ml)**	5.71 ± 2.82	7.03 ± 3.43	5.98 ± 2.84	0.22 ${}^{b}$	-
** SHBG (nmol/ml)**	10.53 ± 6.61 ${}^{c}$	11.51 ± 5.77 ${}^{k}$	22.09 ± 6.24 ${}^{e}$	< 0.001 ${}^{a}$	c & e (p < 0.001) k & e (p < 0.001)
** Tt (ng/ml)**	2.58 ± 1.08 ${}^{c}$	3.30 ± 0.92 ${}^{d}$	5.87 ± 2.08 ${}^{e}$	< 0.001 ${}^{b}$	c & d & e (p < 0.001)
** E2 (pg/ml)**	30.17 ± 13.27 ${}^{c}$	29.26 ± 14.28 ${}^{g}$	21.58 ± 8.70 ${}^{f}$	0.01 ${}^{b}$	c & f (p = 0.01) g & f (p < 0.001)
** Tt/E2 ratio**	0.09 ± 0.04 ${}^{c}$	0.13 ± 0.06 ${}^{d}$	0.30 ± 0.14 ${}^{e}$	< 0.001 ${}^{A}$	c & d (p < 0.001) c & e (p < 0.001) d & e (p < 0.001)
** Tt/LH ratio**	0.41 ± 0.35 ${}^{c}$	0.52 ± 0.43 ${}^{h}$	1.57 ± 1.43 ${}^{e}$	< 0.001 ${}^{b}$	c & e (p < 0.001) h & e (p < 0.001)
** FAI% (Tt/SHBG. 100)**	34.84 ± 25.08	36.77 ± 22.46	29.03 ± 13.80	0.41 ${}^{b}$	-
Data presented as Mean ± SD. 25(OH)D: 25-hydroxy vitamin D, LH: Luteinizing hormone, FSH: Follicle-stimulating hormone, SHBG: Sex hormone-binding globulin, Tt: Testosterone, E2: Estradiol, FAI: Free androgen index. Differences between the 3 groups were tested using one-way analysis of variance (ANOVA) (a), Welch and Brown-Forsythe (A), Kruskal-Wallis test (b). Post hoc tests for normal distribution data (a): Least Significant Difference test (LSD), (A): Dunnett T3. Pairwise comparison for non-normal distribution data were tested using Mann-Whitney test (b). P < 0.05 was considered statistically significant					

## 4. Discussion

The results of the present research showed that men with vitamin D deficiency had lower semen volume, lower sperm counts, less motile and progressive spermatozoa, and lower normal sperm morphology, SHBG levels, testosterone, testosterone/estradiol ratio and testosterone/LH ratio; however, they had higher LH and estradiol levels than men with adequate vitamin D levels.

The results concerning the total and/or progressive motility, total sperm number and normal sperm morphology are in line with those of previous studies, which have reported a consistent positive association between serum 25(OH)D levels and total and/or progressive motility, total sperm number and normal sperm morphology (11). However, the possible mechanisms of the effects of vitamin D on male semen quality and reproductive function have not been fully determined yet (1). But, some evidence suggests that its effects may act through a calcium-dependent mechanism (12).

Moreover, some in vitrostudies have suggested other possible mechanisms related to increased sperm motility and the acrosome reaction observed after the incubation of spermatozoa with 25(OH)2D3, including the increased amount of intracellular calcium in human spermatozoa via vitamin D receptor-mediated calcium release from the intracellular calcium storage, and the elevated adenosine triphosphate synthesis via both cyclic adenosine monophosphate and the activity of the protein kinase A pathway (13, 14).

However, no association between vitamin D levels and sperm motility was found in a previous study (15), whereas a negative correlation was found between both low and high levels of vitamin D and semen parameters in young healthy men in another study (3). Some studies have reported a positive relationship between vitamin D and sperm count (4, 11) or morphology (11), while other studies have reported that vitamin D3 is neither related to total sperm count (1) nor sperm morphology (1, 4).

Therefore, the association between vitamin D deficiency and the parameters of semen is still unclear.

The effect of vitamin D on the production of sex hormones remains unknown too. Most studies have reported no significant relationship between serum levels of 25(OH)D and serum total or free testosterone (16), while others have demonstrated a positive relationship with total or free testosterone (17).

In mice fed with a vitamin D deficient diet, testosterone-synthesizing enzymes appeared to be down-regulated in the testes (18). Moreover, in studies conducted on animals, it has been suggested that changes in intracellular calcium homeostasis in Leydig cells may play a role in the regulation of testosterone secretion. Furthermore, it was previously found that the stimulation of testosterone production in Leydig cells is mediated by LH through the increased production of cyclic adenosine monophosphate and intracellular calcium ions. Also, there is evidence for synergistic effects of LH and vitamin D on testosterone synthesis. The addition of vitamin D along with LH has been shown to increase testosterone production compared to the addition of LH alone. Accordingly, vitamin D may be effective in modulating this process via modulating the calcium-dependent LH response. The ratio of testosterone to LH is considered as the best indicator of both sensitivity to LH and function of Leydig cells. (19). In the present study, the vitamin D sufficient group had lower LH levels; however, they had a higher testosterone to LH ratio compared to the groups with insufficient and deficient vitamin D levels. These results support the proposed mechanism for the effects of vitamin D on increasing the sensitivity of Leydig cells to LH and the production of testosterone.

The previous findings on the association between vitamin D and SHBG have been varied as well. In this regard, some studies have found no significant association between 25(OH)D and SHBG (16), while others have reported a positive correlation with SHBG (17). In line with the results of these studies, in our study, men with lower serum 25(OH)D levels also had lower SHBG and testosterone/estradiol ratios compared to the men with sufficient serum 25(OH)D levels.

Aged over 40 yr with an average BMI above 25 and existence metabolic diseases were the probable reasons for positive relationship between serum 25(OH)D and the FAI in some studies (15, 20). While in another study there was no or negatively relationship between serum 25(OH)D levels and FAI. But it was positively correlated with SHBG levels in younger healthy men (15). The reason for inconsistencies between the results of this research and prior studies regarding FAI may be related to the participants of the study, who were infertile men with an average age of 34 yr and with no other underlying diseases.

In terms of the influence of vitamin D on serum estradiol levels, no significant association was observed in some cross-sectional studies conducted on young healthy men (3, 15). However, a negative relationship was found among serum levels of estradiol and 25(OH)D and ionized calcium in several studies (21, 22). As vitamin D suppresses aromatase function in adipose tissue, so it may subsequently lead to a decrease in bioavailable estradiol (9). In accordance with those studies reporting a negative relationship between serum estradiol levels and 25(OH)D, in the present research, serum estradiol levels and the ratio of testosterone/estradiol were lower in the vitamin D sufficient group compared to in the sufficient and deficient vitamin D groups.

This study had some limitations such as a small sample size and the absence of a fertile control group. In addition, the levels of reactive oxygen species, calcium, phosphorus and osteocalcin were not measured in the obtained samples. Moreover, seasonal differences in the levels of 25(OH)D across the groups were not considered in our study.

## 5. Conclusion

In conclusion, our study showed a relationship between vitamin D levels, and semen parameters and sex hormones in Iranian infertile men. However, further studies with larger sample sizes are needed to improve our knowledge on the association between vitamin D and male infertility as well as on the underlying mechanisms.

##  Conflict of Interest

The authors declare that there is no conflict of interest.
